# Platinum drugs in the treatment of non-small-cell lung cancer

**DOI:** 10.1038/sj.bjc.6600540

**Published:** 2002-10-07

**Authors:** J Cosaert, E Quoix

**Affiliations:** AstraZeneca, Mereside, Alderley Park, Macclesfield, Cheshire, SK10 4TG, UK; Service de Pneumologie Lyautey, Hôpitaux Universitaires, 1, Place de l'Hôpital, 67091 Strasbourg, France

**Keywords:** cisplatin, carboplatin, ZD0473, BBR3464, oxaliplatin, non-small-cell lung cancer

## Abstract

The use of chemotherapy is considered standard therapy in patients with locally advanced non-small-cell lung cancer that cannot be treated with radiotherapy and in those with metastatic non-small-cell lung cancer and good performance status. This approach is also accepted in patients with earlier stage disease, when combined with radiotherapy in those with non-resectable locally advanced disease, or in the preoperative setting. Randomised clinical studies and meta-analyses of the literature have confirmed the beneficial survival effect of platinum-based chemotherapy. Cisplatin and carboplatin have been successfully used with other drugs in a wide variety of well-established two-drug combinations while three-drug combinations are still under investigation. Cisplatin and carboplatin use is limited by toxicity and inherent resistance. These considerations have prompted research into new platinum agents, such as the trinuclear platinum agent BBR3464, the platinum complex ZD0473 and oxaliplatin. These compounds could be developed in combination with agents such as paclitaxel, gemcitabine or vinorelbine in patients with advanced and/or refractory solid tumours.

*British Journal of Cancer* (2002) **87**, 825–833. doi:10.1038/sj.bjc.6600540 www.bjcancer.com

© 2002 Cancer Research UK

## 

Lung cancer has the highest mortality rate of any major malignancy in the developed world, causing an estimated 1 million deaths worldwide annually (Abratt, 1995). In the United States alone it has been estimated that 157 400 deaths from lung cancer will occur in 2001 (American Cancer Society (http://www.cancer. org/ (accessed 18 September 2001)). Mortality due to lung cancer exceeds that related to breast, prostate, colorectal and ovarian cancers combined (American Cancer Society, 2001). Approximately 85–90% of cases of lung cancer are attributable to smoking (Bunn *et al*, 1998).

Non-small-cell lung cancer (NSCLC) represents approximately 75–80% of all lung cancer (Abratt, 1995; Bunn *et al*, 1998; Natale, 1998). Fewer than 25% of patients have resectable disease, due to locally advanced or metastatic disease, which does not allow surgery despite improvements in diagnosis and peri- and postoperative care (Bulzebruck *et al*, 1992). Also, comorbidities, mostly linked to tobacco, may prevent patients with potentially resectable disease from receiving surgery. Overall 5-year survival is between 5 and 13%, and varies with the different stages of the disease (Johnson, 1995; Mountain, 1997; Natale, 1998; Breathnach *et al*, 2001).

This review outlines current treatment options for patients with NSCLC with emphasis on the use of platinum-containing regimens. This disease is inherently resistant to chemotherapy and is associated with lower response rates than many other malignancies (Bunn *et al*, 1998; Natale, 1998) and the optimal treatment is yet to be determined (Breathnach *et al*, 2001).

## MANAGEMENT OF NSCLC

Surgery or radiotherapy is the standard option for patients with early stages of NSCLC. Chemotherapy has shown benefit when used alone in patients with stage IV disease, in combination with radiotherapy in patients with locally advanced disease and in the preoperative setting in those with early stages of NSCLC.

### Surgery and primary radiotherapy

Surgery provides the best chance for cure of localised disease. It is therefore the treatment of choice in stages 0, I and II NSCLC (Deslauriers and Gregoire, 2000). With very careful patient selection, surgery may also be used as part of combined modality treatment in stages IIIA and IIIB (T4) disease (Rosell *et al*, 1994; Roth *et al*, 1994; CancerLinksUSA, http://www.cancer101.net (accessed May 26, 2001)) or stage IV disease to remove single metastatic lesions. However, even if surgery is the best treatment possible, the results are still unsatisfactory with a 5-year survival of less than 35%. These results have led clinicians to evaluate combined modalities of treatment including chemotherapy.

Primary radiotherapy (with curative intent) can be considered in patients with inoperable stages I or II of the disease and sufficient pulmonary reserve. Analysis of one randomised and 26 nonrandomised studies in more than 2000 patients receiving radical radiotherapy for stage I or II disease found that 5-year survival rates ranged from 0 to 42% (Rowell and Williams, 2001). Primary radiotherapy used to be the ‘gold standard’ treatment in locally advanced NSCLC.

### Chemotherapy

The poor efficacy and considerable toxicity of chemotherapy caused great pessimism for many years regarding this approach, as only a small impact on survival was observed.

During the 1980s, cisplatin and carboplatin were studied extensively in NCSLC (Bunn, 1989a,b). Randomised trials as well as meta-analyses provided scientific evidence that platinum-based therapy prolonged survival of patients with advanced NSCLC (stage IIIB with pleural effusions and stage IV) and advanced regional NSCLC (non resectable stages IIIA and IIIB disease) (Non-Small Cell Lung Cancer Collaborative Group, 1995). Experience over the past two decades has shown improvements in survival, symptom control and quality of life in patients with NSCLC who receive chemotherapy instead of best supportive care, and chemotherapy is now considered standard treatment in individuals with advanced NSCLC (Splinter, 1990; Non-Small Cell Lung Cancer Collaborative Group, 1995; Bunn and Kelly, 1998; Johnson, 2000; Bahl and Falk, 2001). More recently, platinum-based chemotherapy has shown to be of interest in the neoadjuvant setting, before surgery in patients with resectable stage IIIA (Rosell *et al*, 1994; Roth *et al*, 1998) and stages I to II disease (Depierre *et al*, 2002). Combined therapy with a platinum and taxane before surgery has also shown notable results, with a 1-year survival rate of 85% in patients with stages I to IIIa NCSLC reported after treatment with paclitaxel and carboplatin (Pisters *et al*, 2000). Another large randomised Intergroup trial is ongoing in the United States that is evaluating the efficacy of paclitaxel plus carboplatin in patients with early stage NSCLC.

The first generation agents in NSCLC (cisplatin, mitomycin-C, iphosphamide/cyclophosphamide, vindesine, vinblastine and etoposide) produced response rates ranging from 15 to 25% when used as monotherapy (Bakowski and Crouch, 1983; Grant and Kris, 1995) but, with the exception of cisplatin, had an unclear effect on survival. Second generation agents (gemcitabine, paclitaxel, docetaxel, vinorelbine, irinotecan and topotecan) showed response rates of 20–25% (Bunn *et al*, 1998). Moreover, randomised studies comparing monochemotherapy with paclitaxel, gemcitabine or docetaxel versus best supportive care showed a survival benefit in the chemotherapy arm, (Anderson *et al*, 2000; Ranson *et al*, 2000; Roszkowski *et al*, 2000) emphasising the results of the meta-analysis of the NSCLCCOG (Non-Small Cell Lung Cancer Collaborative Group, 1995). Additional reports of large randomised trials (one a pooled analysis of two trials), each conducted in more than 700 patients, have confirmed the survival benefit of cisplatin-based combined two- or three-agent chemotherapy versus best supportive care (Cullen *et al*, 1999; Stephens *et al*, 2002).

### Cisplatin and carboplatin

Platinum agents have currently shown the greatest promise in patients with NSCLC. These agents induce their cytotoxic effects by targeting cellular DNA and are active against a number of tumour types (Go and Adjei, 1999). Cisplatin is thought to act by activating apoptosis and altering a number of other cellular parameters. It forms adducts with all DNA bases but preferentially binds to the *N*^7^ positions of guanine and adenine in intact DNA. The main DNA lesions produced by both cisplatin and carboplatin, accounting for a total of 95% of platinum-DNA adducts, are at the G-G, A-G and G-X-G intrastrand crosslinks (Fink and Howell, 2000).

The dosages at which these agents are given varies according to the agent(s) with which they are being combined and the status of the patient. However, cisplatin is usually given at a dosage of 50–120 mg m^−2^ per cycle, whereas the dose of carboplatin is usually customised for each patient using the area under the concentration-time curve (AUC) and renal function of the patient (Calvert *et al*, 1989; Chatelut *et al*, 1995), because this drug undergoes extensive renal excretion. An AUC of 4–6 per cycle, which is approximately equivalent to a dose in the range 200–350 mg m^−2^ per cycle, is usual. Both platinum agents are usually given every 3–4 weeks, according to the haematological status of the patient, for 3–6 cycles.

Analysis of the Southwest Oncology Group (SWOG) database of 2531 patients with extensive NSCLC (1974–1988) showed the use of cisplatin to be an independent predictor of improved outcome (Albain *et al*, 1991). Thus, most clinical studies of chemotherapy in advanced or locoregionally advanced NSCLC in recent years have incorporated cisplatin. However, because of the toxicity of cisplatin (see below), less toxic platinum alternatives have been developed. The most extensively evaluated has been carboplatin (Bunn, 1989b), with studies demonstrating the efficacy of carboplatin, alone (Bonomi *et al*, 1989; Bunn, 1989a,b; Gatzemeier *et al*, 1990a; Kreisman *et al*, 1990) or in combination (Gatzemeier *et al*, 1990b). The available data suggest that carboplatin can substitute cisplatin in patients with stage IIIB/IV NSCLC (Lokich and Anderson, 1998; Go and Adjei, 1999; Zatloukal *et al*, 2001). However, direct comparisons between cisplatin- and carboplatin-based chemotherapy have been very rare (Klastersky *et al*, 1990; Gatzemeier *et al*, 1999). Rodriguez *et al* (2001) presented the results of a randomised study comparing docetaxel plus cisplatin or carboplatin versus vinorelbine plus cisplatin at the 2001 meeting of the American Society of Clinical Oncology. Although the study was not designed to compare carboplatin with cisplatin, results in the carboplatin arm were inferior.

*Two drug combinations* Two types of trials have been conducted to compare monochemotherapy with cisplatin-containing two agent chemotherapy: comparisons with cisplatin monotherapy and comparisons using monotherapy with the non-platinum agent. The relative benefits of combination therapy over monotherapy, shown in many publications, resulted in combination therapy becoming recognised standard practice (Splinter, 1990; Marino *et al*, 1995; Lilenbaum *et al*, 1998) and a number of phase III studies are currently underway or completed that investigate the relative efficacies of several new platinum-containing two-agent combination regimens (Table 1

**Table 1:**
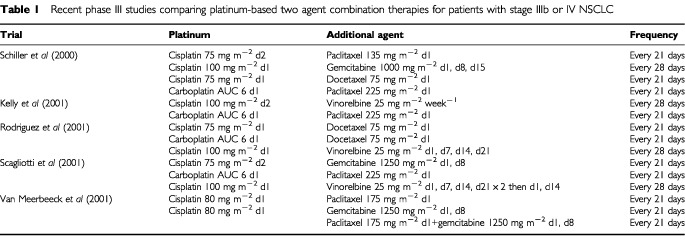
Recent phase III studies comparing platinum-based two agent combination therapies for patients with stage IIIb or IV NSCLC

) (Kelly *et al*, 2001; Rodriguez *et al*, 2001; Scagliotti *et al*, 2001; van Meerbeeck *et al*, 2001; Schiller *et al*, 2002).

Of note, Schiller *et al* (2002) compared cisplatin plus paclitaxel (the ECOG standard of care) with the new combination regimens of cisplatin plus gemcitabine or docetaxel and paclitaxel plus carboplatin (four-arm study). No major differences were observed in terms of efficacy (objective response rate and survival) or toxicity. Similar findings were reported in a trial comparing paclitaxel plus carboplatin with vinorelbine plus cisplatin (Kelly *et al*, 2001).

#### Comparisons between cisplatin containing double therapy and monotherapy with the non-platinum agent

Results of trials comparing monotherapy with vindesine (Elliott *et al*, 1984; Einhorn *et al*, 1986), etoposide (Rosso *et al*, 1990), teniposide (Splinter *et al*, 1996), and vinorelbine (Depierre *et al*, 1994; Le Chevalier *et al*, 1994) with the respective agent combined with cisplatin showed consistently higher response rates in the combination therapy arm, but only about half showed a survival benefit for the combination (Table 2

**Table 2:**
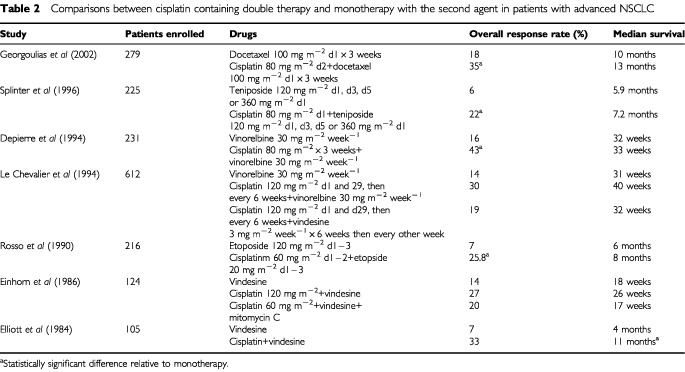
Comparisons between cisplatin containing double therapy and monotherapy with the second agent in patients with advanced NSCLC

). Similarly, preliminary analysis of a multicenter phase III trial comparing docetaxel *vs* docetaxel plus cisplatin in patients with inoperable advanced and metastatic NSCLC showed no survival advantage but a significant improvement in objective response rate with combination therapy (Georgoulias *et al*, 2002; Table 2).

#### Comparisons between cisplatin containing double therapy and cisplatin monotherapy

Comparisons of cisplatin monotherapy and combination therapy with cisplatin plus vindesine (Kawahara *et al*, 1991), etoposide (Klastersky *et al*, 1989; Crino *et al*, 1990), vinorelbine (Wozniak *et al*, 1998), paclitaxel (Gatzemeier *et al*, 2000), gemcitabine (Sandler *et al*, 2000) and tirapazamine (von Pawel *et al*, 2000) consistently showed a higher response rate in the combination therapy arm, but again only half of the trials showed a survival benefit for the combination therapy arm (Table 3

**Table 3:**
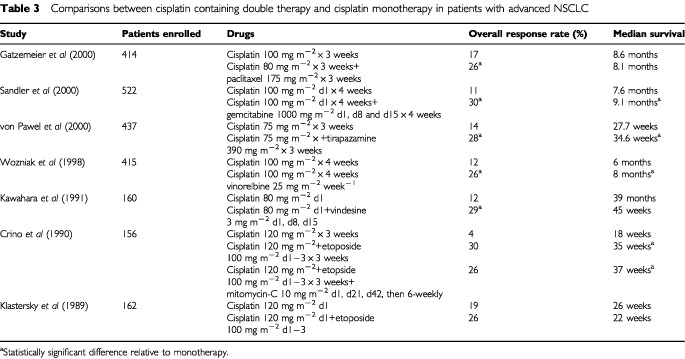
Comparisons between cisplatin containing double therapy and cisplatin monotherapy in patients with advanced NSCLC

).

#### Comparisons between carboplatin containing double therapy and monotherapy with the non-platinum agent

A comparison between monotherapy with paclitaxel and paclitaxel plus carboplatin in 584 patients with advanced NSCLC showed a significant advantage in terms of objective response rate (16 *vs* 30%, *P*<0.0001) and survival distribution (6.5 *vs* 8.5 months, *P*=0.023) in favour of combination therapy but no significant difference between treatments in 1-year survival rate (31 *vs* 36%) (Lilenbaum *et al*, 2002). Similarly, a comparison of gemcitabine with gemcitabine plus carboplatin in 275 patients with advanced NSCLC showed higher objective response rates (12 *vs* 30%) and a significantly longer time to progression (4 *vs* 6 months, *P*=0.001) with combination therapy; the median survival was 9 months for the whole study population (Sederholm, 2002).

#### Conclusions

The survival results reported to date suggest that the importance of inclusion of a platinum agent in the combination therapy setting is still at least open for discussion, although it appears to be accepted that two-agent combination therapy is better than monotherapy.

#### Three drug combinations

No statistically significant survival difference has been observed between regimens containing cisplatin in combination with doxorubicin and cyclophosphamide (CAP), doxorubicin and 5-fluorouracil (AFP), cyclophosphamide and bleomycin (CBP), vindesine (VP), etoposide (EP), or vindesine and mitomycin-C (MVP). Median survival ranged from 21.6 to 26.6 weeks. The MVP regimen showed a trend towards a higher response rate than the other regimens in certain trials with no benefit on survival (Ruckdeschel *et al*, 1985). MVP showed superiority to EP in another trial (Ginopoulos *et al*, 1997). A recent trial that compared triple therapy with cisplatin plus ifosfamide plus mitomycin (MIP) with gemcitabine plus cisplatin showed a higher response rate in the ‘modern’ double therapy regimen and no difference in survival between the two arms (Crino *et al*, 1999). Other comparisons between double and triple therapy with modern drugs did not show any advantage for triple therapy over double therapy (Alberola *et al*, 2001; Souquet *et al*, 2001).

### Regimens containing oxaliplatin

Three small studies are underway to assess combinations of oxaliplatin and gemcitabine (Franciosi *et al*, 2001), paclitaxel (Hoffman *et al*, 2001) or vinorelbine (Monnet *et al*, 2002) in patients with advanced NSCLC. Early results in 24 previously untreated (Hoffman *et al*, 2001), 28 previously untreated (Monnet *et al*, 2002) and 10 previously treated (Franciosi *et al*, 2001) patients show response rates of 25, 35 and 30%, respectively. Oxaliplatin monotherapy has also demonstrated activity in a small study of 33 patients with poor-prognosis NSCLC (Monnet *et al*, 1998).

### Other chemotherapy options

In addition, combinations of paclitaxel or docetaxel with nonplatinum agents such as gemcitabine have shown promising results (Douillard *et al*, 2001b; Georgoulias *et al*, 2001). Indeed, such combinations may be an option for patients unable to tolerate platinum agents or those with compromised performance status. In addition, patients with a performance status of 2 do not benefit from platinum-based chemotherapy (Soria *et al*, 2001). In general, studies comparing non-platinum regimens with platinum-based regimens are still ongoing. In one that is published (Georgoulias *et al*, 2001), no significant difference was seen between gemcitabine plus docetaxel and cisplatin plus docetaxel. The results of such trials need to be confirmed.

Several of the newer agents have been studied as second line chemotherapy in patients with NSCLC and have shown some efficacy (Socinski and Langer, 1999; Huisman *et al*, 2000; Miller and Kris, 2000), especially docetaxel for which there has been two randomised studies (Fossella, 1999a,b; Shepherd *et al*, 2000).

### Combined modality and adjuvant therapy

The use of platinum-based chemotherapy in conjunction with radiotherapy in patients with locally advanced unresectable NSCLC has become standard since the studies of Le Chevalier *et al* (1991) and Dillman *et al* (1990). The NSCLCCG meta-analysis confirmed the survival benefit provided by giving cisplatin-based chemotherapy before radiotherapy over radiotherapy alone (Non-Small Cell Lung Cancer Collaborative Group, 1995). Although it is standard to use induction chemotherapy followed by radiotherapy, there are some arguments favouring concurrent chemoradiation using chemotherapy at systemic dosages (Eberhardt *et al*, 1998; Jeremic *et al*, 1999) or at radiosensitising dosages (Trovo *et al*, 1992; Schaake-Koning *et al*, 1994; Bardet *et al*, 1997; Clamon *et al*, 1999). These two different treatment modalities have been studied in a number of promising phase II trials but there are very limited data from positive randomised phase III trials (Schaake-Koning *et al*, 1994; Furuse *et al*, 1999). Results of these phase III studies support the use of concurrent chemotherapy and radiotherapy in preference to radiotherapy alone (Schaake-Koning *et al*, 1994) or sequential chemotherapy then radiotherapy (Furuse *et al*, 1999).

### Problems with currently used platinum drugs

#### Toxicity

Severe adverse effects limit the use of cisplatin (Mckeage, 1995). Nephrotoxicity may be reduced but not suppressed by hyper-hydration (Hamilton *et al*, 1989; Bissett *et al*, 1990). However, this hyper-hydration is not possible in patients with congestive heart failure, a condition that is not rare in patients with NSCLC. Cisplatin is also one of the most emetogenic drugs used, with considerable variability between individuals. Systematic use of serotonin antagonists has improved control of acute emesis but not delayed emesis (Fauser *et al*, 1999; Gralla *et al*, 1999). Anemia can also occur during treatment with cisplatin. This can be due to several mechanisms, including depletion of intrinsic erythropoietin production (caused by peritubular renal cell depletion), reduced bone marrow stem cell activity and the absence of the stem cell reaction of administered erythropoietin (Dufour *et al*, 1990; Canpolat *et al*, 1994; Wood and Hrushesky, 1995).

Nephrotoxicity and neurotoxicity have been considerably reduced by replacing cisplatin with carboplatin, which shows nephrotoxicity only when used in high dosages. Carboplatin, however, causes dose-limiting myelosuppression (Mckeage, 1995; Bunn, 1989b; ; Judson and Kelland, 2000). Transient rises in bilirubin levels have also been observed (Fields *et al*, 1995).

#### Resistance

Kelland (2000) and Giaccone (2000) reviewed recently in detail the inherent resistance of NSCLC to current platinums. NCSLC is inherently resistant to treatment with cisplatin (Giaccone, 2000), so an understanding of the mechanisms behind this could help to improve the prognosis of many patients with the cancer. Thus, resistance to cisplatin has been studied extensively *in vitro*. A number of resistance mechanisms have been identified including: (a) increased repair of platinum-induced DNA damage (increased nucleotide excision repair or loss of DNA mismatch repair); (b) glutathione or metallothionein drug deactivation; (c) reduced cellular uptake of the platinum; (d) altered apoptosis (Kelland, 2000).

The clinical relevance of these mechanisms is currently not entirely clear; however, tumour cell overexpression of metallothionein has been shown to correlate with chemo-resistance and prognosis in patients with oesophageal and urothelial cancer (Go and Adjei, 1999). Similarly, clinical trials have shown that prognosis is related to lung resistance-related protein abnormalities, which may alter transport of cisplatin; increased repair of cisplatin-DNA adducts; and loss of mismatch repair (Fink and Howell, 2000; Giaccone, 2000). Nucleotide excision repair appears to be the most important pathway for cisplatin-DNA damage, and the critical gene appears to be excision repair cross-complementing (ERCC1) (Giaccone, 2000). A number of studies have shown that high levels of the ERCC1 relative messenger RNA are associated with response and survival after cisplatin treatment (Giaccone, 2000; Rosell and Felip, 2001). Another genetic abnormality though to be related to cisplatin resistance affects the apoptosis gene p53; 60% of NSCLC patients have p53 mutations (Giaccone, 2000). Resistance to carboplatin is less well studied, but it is assumed that similar mechanisms are involved (Go and Adjei, 1999). The pharmacogenomics of these agents is therefore being intensively studied and may dictate therapy choices in the future.

### New platinum agents

The problems associated with the use of current platinum agents, and the need to improve response and survival in patients with NSCLC (and other cancers), have prompted research into new platinum agents that have improved toxicity profiles, may circumvent resistance mechanisms, and have administration schedules that are acceptable to physicians and patients.

New agents include nedaplatin, a cisplatin-like compound registered in Japan and active in NSCLC (Judson *et al*, 1997), and satraplatin, an orally available drug with dose-limiting toxicity similar to that of carboplatin currently being explored in prostate cancer. Two other novel agents, BBR3464 and ZD0473, have shown good results in preclinical and *in vitro* studies, and have potential in the treatment of solid tumours (Judson and Kelland, 2000).

#### BBR3464

BBR3464 is a trinuclear platinum complex that binds to DNA more rapidly than cisplatin and forms long-range interstrand and intrastrand crosslinks. Phase I studies show diarrhoea and neutropenia to be dose-limiting toxicities, without significant nephro-, neuro- or pulmonary toxicity (Calvert *et al*, 1999; Sessa *et al*, 2000). Antitumour activity was observed in colorectal and pancreatic cancer patients after a one-hour infusion of 1.1 mg m^−2^ every 28 days (Calvert *et al*, 1999). A second study (Sessa *et al*, 2000) showed similar toxicity (0.03–0.17 mg m^−2^ day^−1^ for 5 days, repeated every 28 days), in patients with solid tumours unresponsive to previous antitumour treatment. Phase II trials are currently underway.

#### ZD0473

ZD0473 is a new-generation platinum agent designed to deliver an extended spectrum of antitumour activity and overcome platinum resistance mechanisms. A common mechanism of resistance is the replacement of the platinum centre by a thiol moiety. This substitution is hindered by increasing the steric bulk of the molecule, and ZD0473, with its methyl-substituted pyridine side chain, was designed with this property in mind (Holford *et al*, 1998b).

Biochemical studies show that ZD0473 at least partially overcomes mechanisms of inherent or acquired resistance (Holford *et al*, 1998a), and preclinical work indicates activity against cell lines resistant to older platinum agents (Raynaud *et al*, 1997). In man, dose-limiting toxicity is myelosuppression, particularly in patients previously treated with carboplatin (Trigo *et al*, 1999; Hoctin-Boes *et al*, 2001); without evidence of clinically relevant neurotoxicity, nephrotoxicity or ototoxicity when given at doses of 120 or 150 mg m^−2^ (Hoctin-Boes *et al*, 2001).

Of the newer platinum agents, the new-generation agent ZD0473 could be of interest in NSCLC, with good tolerability having been reported in phase I trials in which the drug has been given in combination with paclitaxel, gemcitabine or vinorelbine in patients with advanced and/or refractory solid tumours (Table 4

**Table 4:**
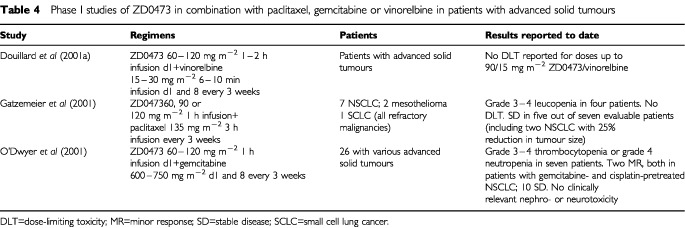
Phase I studies of ZD0473 in combination with paclitaxel, gemcitabine or vinorelbine in patients with advanced solid tumours

). These trials are ongoing, as are phase II monotherapy studies of first- and second-line treatment in patients with NSCLC in which ZD0473 is being given at a dosage of 120–150 mg m^−2^ every 3 weeks.

## CONCLUSIONS

Chemotherapy is now broadly accepted in stage IIIB/IV NSCLC, and there is growing interest in its use in earlier disease when combined with other (local) therapy. Platinum drugs are still considered of crucial interest based on clinical studies and the results of meta-analyses, with the inconvenience of the observed toxicity and the inherent resistance. These observations have prompted the development of second generation drugs and newer platinums (oxaliplatin, BBR3464, ZD0473) and any relative benefits for these approaches will be investigated in the ongoing trials.
